# The Physical Knowledge

**Published:** 1863-01

**Authors:** S. Conant Foster

**Affiliations:** New York Academy of Medicine


THE PHYSICIAN'S KNOWLEDGE—*Anniversary Address by S. Conant Foster*, M. D., *New York Academy of Medicine,—Nov.*, 11., '62.— On the enunciation of the truth that "knowledge is power" no one stops to analyse it except perhaps mentally; but when we come to answer the question—What is knowledge? we shall see that it is not any exercise of the imagination; not alone what is gained by the perceptive faculties. Theories do not constitute knowledge, which is power. True knowledge is something which is certain, positive, the prophet and interpreter of things to come. Knowledge is gained with special effort. A man of general knowledge is seldom in error in his statements. General knowledge is general power. Special knowledge enables one to accomplish what no one without such knowledge could do successfully, as in medicine or the arts. Special knowledge acts upon material objects; general knowledge acts upon the mind.

What is the nature of the physician's knowledge? The ideal physician is a man habituated to careful study; to doubt everything which is not recommended to his understanding by demonstrative evidence. He is accustemed to compare the results of his own observation with the observations of others. He is to exert a controlling influence over the forces of nature. He is the appointed administrator of the ordinances of the Creator.

In comparing physic with law, we find that in the latter there is a certain ebb and flow of human justice ; the sphere of legal knowledge is inevitably narrow, and its tendency is to contract the mind. Theology is more enlarged in its scope, but is enveloped in a considerable mystery. Its positive knowledge is limited to what is revealed. A clergyman's usefulness is not unfrequently limited by his personal influence upon his heares. Medicine has to deal with matter of fact. New laws are continually discovered ; new scientific truths are continually developed.

A great effort has been made of late years to popularize scientific knowledge. Physicians have taken the lead in the measure. There are few physicians at this day who would be unwilling to explain the modus operandi of their remedies, and the reason they have ever been unwilling to do so is on account of the popular ignorance of medical science. To be understood they would have to teach first a little anatomy, a little physiology, a little materia medica, a little chemistry, etc. A physician must possess good health of both body and mind. Though he might be able to prescribe for others he should not prescribe for himself.

Notions of therapeutics are very widely spread. A knowledge of the anæsthetic properties of chlorform has penetrated to the utmost parts of the globe. So of quinine. It becomes the interest as it is the pleasure of the man of true science, to lend his aid to the diffusion of true knowledge.

The knowledge of a law is the result of repeated experiments. The moment one begins to talk of hypothesis it begins to be doubted. The true way is to combine theory with practice. To be a good theorist one must be a good observer; he must not overlook half he sees, nor set down more than he sees, nor throw his observations into a confused mass. Infallibility is an attribute only of Divinity. Errors may become most instructive.

As the public mind becomes more enlightened there is less tendency to ignore scientific facts. In former times there was a difference in scenic representation between the scientific physician and the charlatan. I would extend the great work of educating the people in medical science. "A *little* learning is a dangerous thing," I would give the people *much.* Let them learn what they can of anatomy, physiology, chemistry, etc. While the telescope reveals the stars, let the microscope reveal the atoms of matter.

The relation of the physician to his patient is an interesting and important one. He arrives, examines his patient's pulse, tongue, etc., writes his prescription, gives a few directions as to diet, and leaves. One asks, "what does it amount to?" If a Croton pipe had burst, and you had sent for a plumber, you would have *seen* his work at a glance. The physician during his brief visit has done much. He recognizes the form of the disease as he would the features of a familiar friend. He gets the history of the case, and perhaps attributes the disease to some unsuspected cause.

It must not be forgotten that nature always attempts to cure the patient, and remembring this, the physician must select from the whole range of nature the remedy best calculated to aid her in her efforts; he must consider the time and quantity to administer; he must also employ such moral agencies as he may be able to command. All this the physician must bring to bear in every case he examines; an amount of knowledge not required by any other profession; a knowledge which every physician *must* have who excels. In America the physician may have a certain practical sagacity which will make up much of the deficiency of the thorough knowledge which is required in the old country. Our country should be more glorious in the future than in the past, and civilzation will keep pace with liberty. The practical object of medical science is to relieve suffering humanity, and he who practices it will have his susceptibilities quickened; he will not seek the high places, but will be content with the delight he feels in the possession of knowlege which benefits his fellow man. The profession need not to go about begging recognition, but should go on patiently rearing that grand temple whose foundation was laid centuries ago.

